# Triggers of the Postural Display of Courtship in *Drosophila persimilis* Flies

**DOI:** 10.1007/s10905-017-9641-1

**Published:** 2017-10-18

**Authors:** Mónica Vega Hernández, Caroline Cecile Gabrielle Fabre

**Affiliations:** Department of Zoology, University of Cambridge, Downing Street, Cambridge, CB2 3EJ UK

**Keywords:** *Drosophila*, *persimilis*, *pseudoobscura*, courtship, behaviour, biotremology, tremulation, competition, receptivity, rejection, feeding, copulation

## Abstract

*D. persimilis* courtship shows some flexibility and courting males sometimes perform an elaborate postural display in addition to the standard courtship behaviours shared by most *Drosophila* species. This postural display includes the acrobatic contortion and tremulation of their abdomen, accompanied by the generation of substrate-borne vibrations, and they proffer a nutritional droplet to the female. Here, we use courtship and choice assays to ask what triggers this display and what advantages males may gain from it during courtship. In pair assays, we found no differences in the courtship duration and copulation success between displaying and non-displaying males. In trio assays, however, the female always mated with the male who performed the display. To investigate what promotes the male display, we varied the level of receptivity of the female and studied the impact of a second male. We found that rejection by the female does not induce the male to display, contrary to what was previously suggested. We present evidence that the male display is in fact promoted by the presence of an attentive and sexually receptive female and the absence of male competition, with the greatest exhibition rate obtained if the courted female is starved. These findings provide valuable information about the social ecology of flies, and how internal and external cues influence sexual behaviours and mate choice.

**Electronic supplementary material:**

The online version of this article (10.1007/s10905-017-9641-1) contains supplementary material, which is available to authorized users.

## Introduction

In many animals, courtship behaviours are important traits for reproductive success (Andersson 1994). Sexual selection has influenced the complexity of the courtship displays that a variety of animals use to attract a mating partner and advertise their desire to mate [see for example (Pruett-Jones and Pruett-Jones 1990; Frith and Beehler 1998). Darwin implied that this complexity is a consequence of the “constantly recurring struggle between the males for the possession of the females” with constraints that include direct competition between males (intrasexual selection) and the choice of a mate by the females (intersexual selection). This has sometimes led to complex behaviour patterns of male courtship in some species. Examples of spectacular displays are found in birds and mammals [see for example (Cooper and Forshaw 1977; Clutton-Brock and Albon 1979; Frith and Beehler 1998; Scholes 2006)], as well as arthropods, including some species of flies (Brown 1964, 1965; Spieth 1952, 1966, 1981, 1987; Kaneshiro 1983; Setoguchi et al. 2014; Hernandez and Fabre 2016).

In most *Drosophila* flies, the males display “standard” courtship behaviours that often show only subtle variations between species. The males follow the females, they flutter one wing to produce a species-specific song, they gather gustatory information by licking and tapping the female, they tremulate the abdomen up-and-down to produce substrate-borne vibrational signals and finally they curve the abdomen and attempt copulation (Spieth 1952; Ewing and Bennet-Clark 1968; Fabre et al. 2012; Bontonou and Wicker-Thomas 2014). Males of the species *Drosophila persimilis* perform most of these standard behaviours but they are also one of the few *Drosophila* species in which males sometimes exhibit an additional postural display of high complexity (Brown 1965; Spieth 1981; Kaneshiro 1983; Setoguchi et al. 2014; Hernandez and Fabre 2016), which we call the postural display of courtship (PDC). The PDC is about 20 s long and the male usually performs it twice to the female during his 2 min long courtship (Hernandez and Fabre 2016); the PDC includes the acrobatic contortion and tremulation of the male abdomen, the production of substrate-borne vibratory signals, the upwards movement of the wings as well as the offering of a droplet to the female (Brown 1964, 1965; Hernandez and Fabre 2016). We previously reported that the PDC is in fact exhibited in only ~ half of the *D. persimilis* courting pairs (the other half relying exclusively on the standard courtship behaviours) but it is not clear why this is the case and which contexts may favour the PDC exhibition in males (Hernandez and Fabre 2016). This flexibility in the male behaviour was also surprising because *Drosophila* courtship is usually described as being largely stereotyped [see for example (Spieth 1952; Greenspan and Ferveur 2000)] and, to our knowledge, the finding that ~ half of the *D. persimilis* courting pairs behave differently to the other half had not previously been reported.

In most animals performing complex courtship displays, it is difficult to analyse the effect of social interactions and ask how a particular behaviour is promoted or inhibited depending on context. Researchers therefore rely mostly on prolonged observations in the wild or in enclosures and happenstance [see for example (Kavanau 1963; Lehner 1998; Whitehead 2008; Duvall et al. 2012; Suen and Ary 2014). Unlike birds and mammals, however, large populations of flies can easily be maintained in the laboratory and experiments assessing various courtship contexts in controlled environments can be undertaken, video-monitored and analysed (Anholt and Mackay 2004; Ejima and Griffith 2007; Nichols et al. 2012). Such behavioural studies may provide additional and valuable information about the social ecology of flies (Markow and O’grady 2008; Markow 2015). Here, to understand why the PDC of *D. persimilis* flies is not observed in all the courting pairs, we asked: 1) Does the PDC promote copulation success? 2) Does the reproductive and nutritional status of females, and their associated behavioural cues, influence the exhibition of the PDC by males? 3) How may conditions such as the presence of a rival male influence the PDC?

## Methods

### Flies

*Drosophila persimilis (*UC San Diego Drosophila stock center, stock number 14011–0111.00, collected from Cold Creek, California*), D. pseudoobscura* (UC San Diego Drosophila stock center, stock number 14011–0121.00, collected from Tucson, Arizona) were raised on standard wheatmeal medium under a 12:12 h light:dark cycle and kept at 23 °C with 65% humidity. Adult flies were collected upon eclosion with light CO2 anaesthesia. Before mating, individual males or small groups of ten virgin females were kept in vials with similar amount of fresh food and were tested at similar ages after hatching. This way, we aimed for animals tested to have absorbed similar amounts of food before performing treatments and experiments. Filming of courting pairs were performed at a temperature of around 23 °C.

### Behavioural Recording of Courtship Assays

Pairs of flies were tested at 7 days old when they are most active in courtship. Their behaviour was recorded with a 100 mm macro lens and a Stingray F-033B camera (Allied Vision Technologies; Stadtroda, Germany) and acquired with the Astro IIDC (Aupperle Services and Contracting; Calgary, Canada) or the Debut Video Capture (Pro Edition) softwares into a laptop computer. Flies were filmed in transparent plexiglass courtship chambers (10 mm diameter and 9 mm height). Recording was started at the initiation of courtship and for approximately 600 s, or until copulation occurred. Each pair was tested only once. Before each test, chambers were washed with ethanol and dried.

### Behaviour Annotations and Analysis

Movies were annotated with the Annotation software (Peter Brodsky, version 1.3), registering courtship, copulation, moving and vibrating (i.e. “fluttering”) the wings (a standard male courting behaviour) and the exhibition of the postural display (that includes the wing-posture, movements and tremulation of the abdomen, movements of the head and legs, production of liquid droplets, etc.), and also whether the female was extruding the ovipositor. The data for each movie were imported into Excel files. We generated the box plots using the R program -BoxPlotR- from the Tyers Lab (http://boxplot.tyerslab.com/). The box limits specify the 25th and 75th percentiles. Bold middle lines indicate medians, and crosses indicate the means. Chi-square tests were used for comparing the display of PDC and no PDC, as well as copulation successes (*Χ* ^2^_2_ and *P* values are provided, with significant results given at *P* < 0.05) and we calculated the error intervals for binomial experiments for two standard deviations’ accuracy (95% confidence). Mann-Whitney U tests (two-tailed) were used for the comparisons of courtship duration, wing fluttering and ovipositor extrusions (values of *U*, *N* and *P* are provided, and significant results are given at *P* < 0.05). Standard errors (± s. e.) of the means are all given for 95% confidence.

### Choice Assays

Female choice assays (used to study the effects on female mate choice and male behaviour) where performed using *D. persimilis* females with either two *D. persimilis* males, or with one *D. persimilis* and one *D. pseudoobscura* male. Flies were six to eight days old. We used 10 × 9 mm plexi-glass filming enclosures. The enclosures had three layers that could be set as separate chambers where each flies could be placed individually. The three layers were fused into one single chamber upon filming. *D. persimilis* and *D. pseudoobscura* are sister species and are impossible to tell apart morphologically on the videos. In order to distinguish the male flies we always initially placed the *D. pseudoobscura* male in the lower chamber layer for easy tracking and identification of the males.

### Starvation of Females

Females were maintained in a tube containing humidified cotton but no food for 48 h before performing the courtship assays.

### Courtship with Mated Females

Virgin females were paired with a male and the pair was observed until they achieved copulation. The mated female was then retrieved and kept in a fresh vial for 48 h after copulation before her pairing with another male was observed.

## Results

### Why Do Only Half of the Courting Pairs Exhibit the Male Postural Display of Courtship (PDC)?

(i) *Comparison of the courtships in which males exhibit the PDC and courtships in which males do not*

**Fig. 1:**
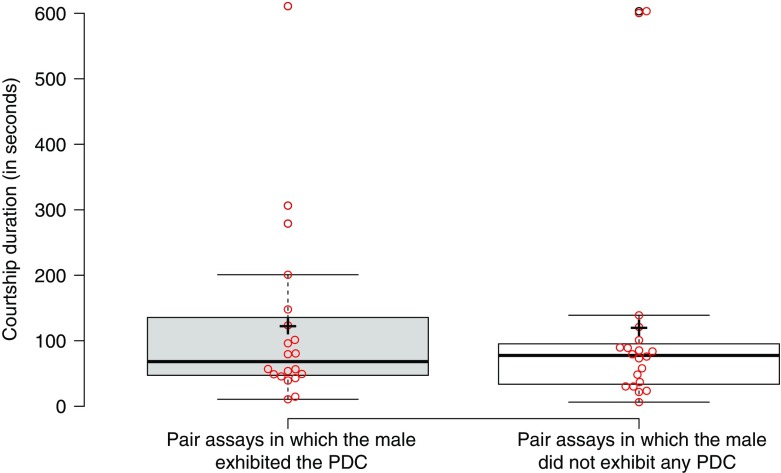
Comparison of the courtship duration of *D. persimilis* in pair assays depending on whether the male exhibits the PDC or not. 20 courting pairs in which males exhibit at least one PDC are compared to 20 courting pairs in which males do not exhibit any PDC. The mean duration of courtship (± s.e.) is 122 ± 31 s for pairs in which the male exhibited the PDC and 120 ± 37 s for pairs in which the male did not exhibit the PDC (*P* = 0.65). All the courting pairs also perform the “standard” courtship parade that includes the movements of wing fluttering

**Fig. 2:**
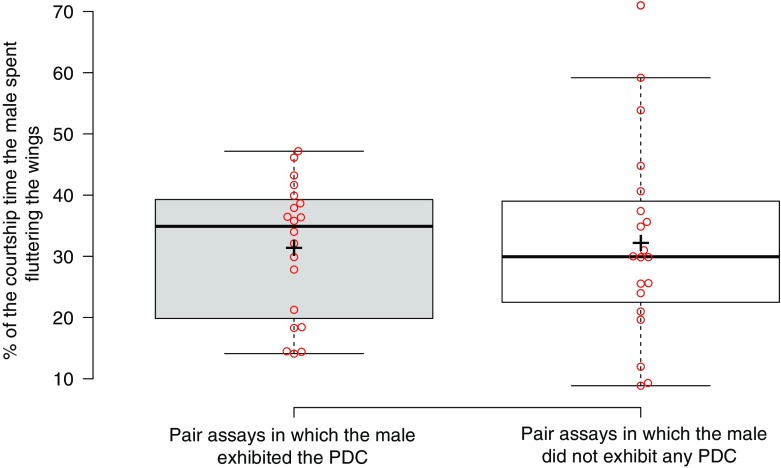
Comparison of the percentage of wing fluttering performed by males during courtship of *D. persimilis* in pair assays depending on whether the male exhibits the PDC or not. 20 courting pairs in which males exhibit at least one PDC are compared to 20 courting pairs in which males do not exhibit any PDC. The mean percentage of wing fluttering during courtship is 31.40 ± 2.45% for pairs in which the male exhibited the PDC and 32.20 ± 3.60% for pairs in which the male did not exhibit the PDC (*P* = 0.69)

We previously reported that courting *D. persimilis* males exhibited the PDC (2.6 ± 0.7 PDCs per courtship; Supplementary Movie S1) in addition to the “standard” courtship behaviours in only ~ half (47.5%) of the courting pairs (*n* = 40 courting pairs; Table 1A and (Hernandez, 2016 #110)), in the other half of the pairs only the standard courtship behaviours were performed (Hernandez and Fabre 2016). We wondered if the males that performed the PDC in pair assays were more likely to copulate with the female. To our surprise, we found no significant difference between the two types of courtship, as in both cases ~90% of males achieved copulation (Table 1A). Similarly there was no difference in the duration of the courtship preceding copulation (Fig. 1; *U* = 183, *N*_*1*_ = *N*_*2*_ = 20, *P* = 0.65) or of other standard courtship behaviours, such as the amount of wing fluttering that produces a species-specific song (Fig. 2; on average ~30% of the courting time was spent wing fluttering in both cases; *U* = 185, *N*_*1*_ = *N*_*2*_ = 20, *P* = 0.69) (Spieth 1952; Waldron 1964; Noor and Aquadro 1998). Thus, PDC does not appear to promote copulation success in paired male-female assays.

(ii) *Is the PDC a response to female’s receptivity and reproductive status?*

**Table 1:** Male PDC behaviour and copulation success depending on the social context during courtship

Type of assay		Percentage of assays showing *D. persimilis* PDC	Copulation success of the *D. persimilis* PDC-displaying males	Copulation success of the *D. persimilis* males that do not display PDC
*A*	Pair assays:	47.5 ± 7.89	90 ± 6.70	90 ± 6.70
*(M)*	*D. persimilis*			
*(F)*	*D. persimilis*			
*B*	Pair assays:	0	N/A	12.5 ± 5.22
*(M)*	*D. persimilis*			
*(F)*	mated *D. persimilis*			
*C*	Choice assays:	16.30 ± 5.63	100	77.80 ± 7.85
*(M)*	*D. persimilis*			
*(M)*	*D. persimilis*			
*(F)*	*D. persimilis*			
*D*	Choice assays:	26 ± 6.47	42 ± 7.27	49 ± 8.41
*(M)*	*D. persimilis*			
*(M)*	*D. pseudoobscura*			
*(F)*	*D. persimilis*			
*E*	Pair assays:	100	83.30 ± 10.76	N/A
*(M)*	*D. persimilis*			
*(F)*	starved *D. persimilis*			

The percentage of assays showing *D. persimilis* PDC, the copulation success of *D. persimilis* males displaying the PDC and the copulation success of *D. persimilis* males that do not display the PDC are shown for **(A)** pair assays including one *D. persimilis* male and one *D. persimilis* (virgin, normally fed) female (*N* = 40), **(B)** pair assays including one *D. persimilis* and one *D. persimilis* mated female (N = 40), **(C)** choice assays including two *D. persimilis* males and one *D. persimilis* (virgin, normally fed) female (*N* = 43), **(D)** choice assays including one *D. persimilis* male, one *D. pseudoobscura* male and one *D. persimilis* (virgin, normally fed) female (*N* = 46), **(E)** pair assays including one *D. persimilis* male and one *D. persimilis* (virgin, starved) female (*N* = 12)

**Fig. 3:**
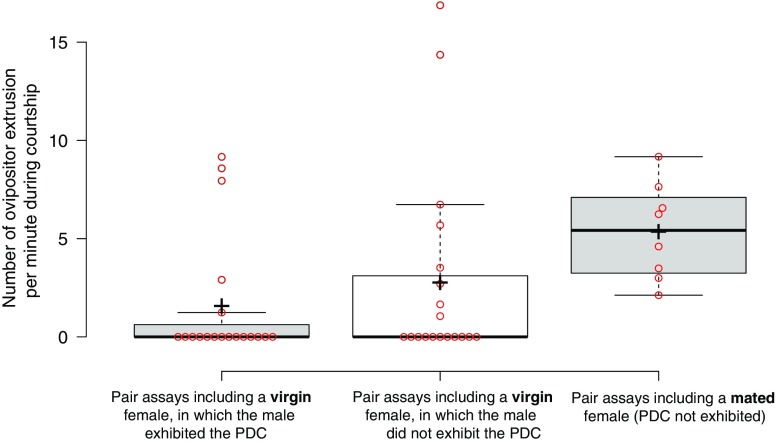
Comparison of the female receptivity during *D. persimilis* courtship in pair assays. In courting pairs in which males exhibit at least one PDC, virgin females perform on average 1.57 ± 0.73 ovipositor extrusions per minute (*N* = 19). In courting pairs in which males do not exhibit any PDC, virgin females perform on average 2.77 ± 1.14 ovipositor extrusions per minute (N = 19). Mann-Whitney statistical test indicates that the number of ovipositor extrusions for these two types of courting pairs is not significantly different (*P* = 0.4). In courting pairs in which the female has previously mated with another male (and no male PDC is observed), the females perform on average 5.35 ± 0.87 ovipositor extrusions per minute (*N* = 8), and this result is significantly different (*P* = 0.01) from the previous pair, at *P <* 0.05

It was unclear from previous studies whether the display might follow rejection by the female (Brown 1964, 1965; Steele 1986a, b). To determine what might drive males to perform such an elaborate display, we analysed the female’s behaviour during courtship. We quantified ovipositor extrusions, an alleged signal of female rejection in *Drosophila* (Spieth 1952; Bastock and Manning 1955; Connolly and Cook 1973). We found that, contrary to previous expectations (Brown 1965), males did not perform the PDC in response to female rejection. On average, virgin females paired with males that did perform the PDC did not extrude their ovipositor significantly more (1.57 ± 0.73 ovipositor extrusions per minute) than virgin females paired with non-displaying males (2.77 ± 1.14 ovipositor extrusions per minute) (Fig. 3; *U* = 154, *N*_*1*_ = *N*_*2*_ = 19, *P* = 0.4), i.e. they rejected the male as much. When we paired *D. persimilis* males with females that had previously mated, courtships were associated with a complete lack of PDC (Table 1B) and copulation success was low (Table 1B; 12.5% on average, compared to 90% in the case of virgin females; *Χ* ^2^_2_ = 41.14, *P* < 0.001). Given that mated *Drosophila* females exhibit much more rejection behaviour than virgin females (Bastock and Manning 1955; Connolly and Cook 1973), which we also observed in *D. persimilis* (Fig. 3; 5.35 ± 0.87 ovipositor extrusions per minute; *U* = 30, *P* < 0.05), our observations suggest that the PDC is not a response to female rejection, but is actually less likely to occur when the female displays rejection behaviours. This is in agreement with our previous observation that the male interrupted his display of the PDC if the female was not attentive to it (Hernandez and Fabre 2016) and suggests that the PDC is more likely to occur if the female is receptive.

(iii) *Is the PDC influenced by intraspecific male-male competition and do females prefer PDC-exhibiting males?*

We performed choice assays where a virgin female was placed in a chamber with two *D. persimilis* males (Table 1C); the behaviours of the three flies were analysed. In all the trios observed, both males displayed the standard courtship behaviours (following the female, wing vibrations, attempted copulations, etc.) (Spieth 1952; Brown 1965; Hernandez and Fabre 2016). They also displayed aggressive behaviour towards each other, such as wing flicking (Brown 1965). These aggressive behaviours disturbed their courtship towards the female (Suppl. Movie S2). In the majority of these choice assays (36/43), neither male displayed PDC (Table 1C). This was surprising because our results from the pair assays, in which 47.5% of males exhibited PDC, suggested that at least one male within a choice assay should display PDC in ~75% of cases. Nevertheless, we observed only 16.30% of choice assays where one of the males displayed PDC (*N* = 43), which is significantly lower (*Χ* ^2^_2_ = 79.837, *P* < 0.001). These results indicate that the presence of another conspecific male deters males from exhibiting the PDC. This was not due to overcrowding, because when the chambers contained one male and two females, 67% of assays contained males that performed the PDC (*N* = 31; 47.5% versus 67% when males are in the presence of one or two females, respectively; Table 1A; *Χ* ^2^_2_ = 3.9, *P* = 0.048).

In the 7/43 two-males one-female choice assays in which one of the males performed the PDC, the female copulated with the PDC-displaying male (i.e. 100% of copulation success for PDC-displaying males; Table 1C). Thus, when they can choose, females prefer males that exhibit the PDC.

(iv) *Is the PDC influenced by interspecific male-male competition?*

*D. persimilis* and *D. pseudoobscura* are sympatric species that do not hybridize in the wild and produce sterile progeny in the laboratory. *D. pseudoobscura* males also perform PDC during their courtship (Brown 1964, 1965; Hernandez and Fabre 2016), and we found that PDC occurred in 52.94% of *D. pseudoobscura* pair assays (*N* = 30). When a *D. persimilis* male was introduced into a chamber with a *D. persimilis* female and a *D. pseudoobscura* male, the *D. persimilis* male performed PDC in only 12/46 cases (i.e. 26% of the assays; Table 1D). This is significantly less than the PDC rate in *D. persimilis* pair assays (*Χ* ^2^_2_ = 8.712, *P* = 0.003), but similar to the results obtained with male-male intraspecific competition (*Χ* ^2^_2_ = 1.27, *P* = 0.26). Thus, *D. persimilis* males are less likely to perform PDC when a *D. pseudoobscura* male is also present. In these choice assays, the copulation success of *D. persimilis* males was low (<50%) whether PDC was displayed or not (Table 1D), probably due to *D. pseudoobscura* male’s active courtship behaviours (Suppl. Movie S3), as was previously described (Noor 1996). Thus, male *D. persimilis* courtship and the disposition of *D. persimilis* females to copulation were disturbed by the presence of another species within the chamber (Suppl. Movie S3).

*D. pseudoobscura* males have been reported to have low ability to discriminate between *D. pseudoobscura* or *D. persimilis* females, the female being the one that chooses ultimately her mating partner (Mayr 1946). Despite their active and aggressive courtship (Suppl. Movie S3), *D. pseudoobscura* male must have sensed female’s low receptivity as only 5/46 *D. pseudoobscura* males displayed the PDC (i.e. 10.86% of the choice assays, compared to 52.94% for *D. pseudoobscura* pair assays; *Χ* ^2^_2_ = 12.71, *P* < 0.001) and only 2/46 *pseudoobscura* males achieved copulation with the *D. persimilis* female (i.e. 4.35% of the assays showed copulation success compared to 88.23% for *D. pseudoobscura* pair assays; *Χ* ^2^_2_ = 44.33, *P* < 0.001).

Thus, the PDC and copulation are less likely to happen if *D. persimilis* and *D. pseudoobscura* males are both courting a *D. persimilis* female, compare to *D. persimilis* intraspecific two male-one female choice assays (Table 1 C, D).

(v) *Is the PDC a response to female’s nutritional status?*

Because the PDC includes a step in which the male feeds the female, we wondered if the male decision to exhibit the PDC might be related to the nutritional status of the female. We paired males with virgin females that had been starved (except for water) for 48 h and observed that the males exhibited the PDC in 100% of pairs (compared to 47.5% when we used fed females; *Χ* ^2^_2_ = 13.532, P < 0.001; Table 1E), suggesting that the PDC is stimulated by the presence of hungry females (Suppl. Movie S4). In these pair assays, the average number of PDCs was 4 ± 0.6 per courtship (*N* = 12). Copulation success of these males was high, similar to that of males in pair assays (Table 1D; *Χ* ^2^_2_ = 0.017, *P* = 0.89). It therefore appears that males receive cues from the female that are related to her hunger, which lead them to perform the PDC; these cues could be directly related to female hunger, or it could be that hungry females simply reject less leading to more PDC. Consistent with this second possibility, we found that the hungry females were very receptive, as none displayed ovipositor extrusion (*N* = 13); this is consistent with previous findings in other *Drosophila* species and in other insects where hungry females are more sexually receptive (Brown 1997; Takakura 2004; Immonen et al. 2009; Lebreton et al. 2015).

## Discussion

It was surprising to observe that, in pair assays, the courtship duration and copulation success of males that did not exhibit the PDC were similar to those of males that did exhibit the PDC. This suggested that exhibiting the PDC did not improve male courtship efficacy. The courtship of insects is rarely undisturbed, however, and a more realistic situation is a context where (at least) one other male is present in the area on which flies aggregate to court. In such a situation it is expected that intraspecific competition between males could strengthen the display of male characteristics that confer mating success (Clutton-Brock and Albon 1979; Andersson 1994; Clutton-Brock and Huchard 2013) and that males exhibiting the PDC might therefore be advantaged over other males. When we generated such a situation, we first found that *D. persimilis* males were less likely to perform the PDC when another male was present. We hypothesise that the reduction in PDC could be due to olfactory or behaviourally inhibitory cues as both males spent considerable amount of time interacting and flicking the wings at each other, which disturbed their focus from courtship. The male that exhibited the PDC in such a context, however, gained a clear advantage and was certain to copulate with the female. In birds, male-male competition also influences female mate choice by reducing the number of males eligible to display to females as interactions among male birds can often take the form of overt aggression or disruption of the display to females (Foster 1983; Trail 1985).

Intersexual signals are important for the male and female partners to regulate their investment during courtship (Thornhill and Alcock 1983). Females may signal their level of receptivity by air-borne and pheromonal cues [See for example in insects: (Waage 1984; Lasbleiz et al. 2006; Maxwell et al. 2010; Wirmer et al. 2010; Bontonou and Wicker-Thomas 2014)]. They may also use movement of their body parts, such as wing movement or ovipositor extrusion as signs of rejection (Connolly and Cook 1973). Males may modulate their courtship in response to these signals from the female (Waage 1984; Patricelli et al. 2002; Lasbleiz et al. 2006). Examples show that this modulation means that males display more or less intense courtship (Patricelli et al. 2002; Guillermo-Ferreira and Bispo 2012). We found that *D. persimilis* females appear to signal their level of receptivity through (at least) ovipositor extrusions. In response to these female cues, *D. persimilis* male may, in turn, display the PDC behaviour. Our observations indicate that they may do so if they are courting receptive and attentive females - we have previously described how males may interrupt their PDC if the female is not attentive to their display (Hernandez and Fabre 2016) - and that they may not display if their wooing is received by female’s rejection behaviours.

We wondered how the PDC display might be affected by interspecific male competition. *D. persimilis* and *D. pseudoobscura* are sympatric species that do not produce hybrids in the wild (Lancefield 1929; Dobzhansky and Epling 1944), but they are morphologically similar, with similar cuticular hydrocarbon patterns (Noor 1996). They differ by genetic divergences, the size of their penis and the courtship songs that they produce (Koopman 1950; Brown 1965; Noor and Aquadro 1998; Hernandez and Fabre 2016). When we promoted situations in which a *D. persimilis* female was in an arena with both a *D. persimilis* male and a *D. pseudoobscura* male, we found that, again, the *D. persimilis* male was less likely to perform the PDC than in pair assays. Again, this might be due to olfactory or behaviourally inhibitory cues between the males (Mayr and Dobzhansky 1945). *D. pseudoobscura* males have been reported to have low ability to discriminate between *D. pseudoobscura* or *D. persimilis* females, the female being the one that chooses her mating partner (Mayr 1946). These reports fit with our observations that the *D. pseudoobscura* males courted the *D. persimilis* female actively, as was previously reported (Mayr 1946), yet exhibited almost complete lack of PDC in these assays, suggesting low female receptivity to their courtship. The lack of PDC by the *D. pseudoobscura* males might further contribute to the isolating mechanisms that ensure sexual isolation between these species. It is unclear, however, how often this situation may happen in nature as these species might court with a preference for different times of the day and at different temperatures (Koopman 1950; Carson 1951; Brown 1965; Noor 1996).

Flexible mating patterns are typical of vertebrates (Lott 1991) while *Drosophila* courtship is considered to be a rather stereotyped series of behaviours (Greenspan and Ferveur 2000). However, *D. persimilis* males appear to adopt flexible strategies to obtain the consent of the females. They do not reliably exhibit the PDC and whether they do depends on the context (presence of other males) and/or the status of the courted female (both reproductive and nutritional). We found previously that the PDC includes substrate-borne vibrations that are very likely, as in *D. melanogaster,* to stop the female moving and to promote copulation (Fabre et al. 2012). The offering of food is also effective in slowing the female down and could help achieve copulation (Hernandez and Fabre 2016). We do not know what is contained in the liquid droplet; it is possible that it may promote egg laying (Steele 1986a, b; Immonen et al. 2009). Because the PDC is exhibited at high frequency to starved females, it is likely to contain at least some nutrients. The flies in this study were laboratory-reared strains and the courtship and feeding behaviour of *D. persimilis* in the wild might differ. Nevertheless, such food offering may relate to the ecology of *D. persimilis* flies. If the pair was meeting and courting on food in the wild, one might argue that offering a droplet of food would make little sense. However, it was shown previously that *D. persimilis* flies feed on a variety of materials in the wild even though oviposition is restricted to other particular sites, leading to the suggestion that there is some separation between “feeding sites” and “breeding sites”. In addition, *D. persimilis* males gather in particular areas (Carson 1951) and this is reminiscent of the lek behaviour of some birds, mammals and a few insects (Shelly and Whittier 2010; Clutton-Brock and Huchard 2013), leks being communal display grounds in which males display and females attend only to mate. *D. persimilis* males might therefore congregate in “hotspots” of female traffic on the way to resources and display the PDC to attract receptive females towards mating sites (Shelly and Whittier 2010; Dobzhansky et al. 2012; Clutton-Brock and Huchard 2013). There may be a pseudo-lek situation in *D. persimilis* flies that could help explain why offering nutritive elements might attract and retain females for mating.

## Electronic supplementary material

Suppl. Movie S1 **A video clip of the PDC exhibited by** ***D. persimilis*** **males to females during courtship.** The video is played first in real time, and then at 3 times slower. The male stands by the female; the male extends his back legs and crouches on his front legs; he raises and then droops the abdomen; the male quivers the abdomen up-and-down; the male spreads his wings and takes up a “wing-posture”; the male moves his middle legs in rowing movements; the male raises one or both of his front legs up and down; the male moves his head downwards; the male extrudes his proboscis; the male produces a liquid droplet; the male feeds the female, directly (in some cases the droplet is left on the ground and the feeding is made indirectly). Video clip from Fabre and Hernandez, 2016. (M4 V 5784 kb)

Suppl. Movie S2 **A video clip showing a choice assay where a** ***D. persimilis*** **virgin female was placed in a chamber with two** ***D. persimilis*** **males.** Both males interact with each other, instead of courting the female. One male follows the other male, taps him with his legs and displays wing flicking to its rival. Then, both males try to follow and court the female with the standard courtship behaviours. The female displays ovipositor extrusions. (M4 V 4506 kb)

Suppl. Movie S3 **A video clip showing a choice assay where a** ***D. persimilis*** **virgin female was placed in a chamber with one** ***D. persimilis*** **male and one** ***D. pseudoobscura*** **male.** The *D. pseudoobscura* male actively courts the female, with aggressive attempted copulations, which the female rejects. He also shows vigorous aggression towards the *D. persimilis* male, including following him, leg tapping and flicking of the wings. During these aggressions, the *D. persimilis* male can court very little (but in the end the *D. persimilis* male successfully copulates with the female; not shown). (M4 V 5622 kb)

Suppl. Movie S4 **A video clip showing a pair assay where a starved** ***D. persimilis*** **virgin female was placed in a chamber with a** ***D. persimilis*** **male.** Early on during courtship the male performs a PDC; the female approaches and feeds onto the droplet. The male then performs wing fluttering, and two other PDCs during which he feeds the female again. The female appears to be receptive (no ovipositor extrusion is displayed and she doesn’t move away from the male). The last display doesn’t appear to lead to feeding of the female but an anal droplet is produced. Copulation is achieved at 53 s. (M4 V 6719 kb)
